# ﻿Two new species of mimetic Pachyrhynchini (Coleoptera, Curculionidae, Entiminae) from Rizal, Luzon Island, Philippines

**DOI:** 10.3897/zookeys.1245.155167

**Published:** 2025-07-15

**Authors:** Analyn A. Cabras, Randel D. Estacio, Daven Jayson D. Agbas, Graden G. Obrial

**Affiliations:** 1 Faculty of Agriculture and Life Sciences, Davao Oriental State University, Dahican, Mati City, 8200 Philippines; 2 Terrestrial Invertebrate Research Laboratory, URESCOM, Davao Oriental State University, URESCOM, Dahican, Mati City, 8200 Philippines; 3 Zoology Division, National Museum of Natural History, Malate, Manila, Philippines; 4 Philippine Coleopterists Society Incorporated, TroGenIL, Davao Oriental State University, Mati City, 8200, Philippines; 5 College of Education, Quezon City University, 673 Quirino Hi-way, San Bartolome, Novaliches, Quezon City, 1116 Philippines; 6 Faculty of Computing, Data Science, Engineering, and Technology, Davao Oriental State University, Mati City, 8200, Philippines; † Deceased

**Keywords:** Endemic, *Eupachyrrhynchus*, Greater Luzon, *Metapocyrtus*, mimicry, snout beetle, weevil

## Abstract

Two new species belonging to the genera *Eupachyrrhynchus* Heller, 1912 and *Metapocyrtus* Heller, 1912—*E.joybelmonteae***sp. nov.** and M. (M.) daraitanensis**sp. nov.**—are described and illustrated from Tanay, Rizal, Luzon, Philippines. Notes on their mimicry complex and ecology are also presented.

## ﻿Introduction

In the Philippines, the tribe Pachyrhynchini is one of the charismatic taxa of beetles, having vibrant and striking colorations and patterns. Currently, it is one of the well-studied groups represented by about 600 species and 18 known genera, 14 of which are endemic to the country, with Luzon Island considered as the biogeographic center of the tribe’s diversity (Cabras et al. 2024; Obrial et al. 2024). One of the endemic genera from Luzon Island is the genus *Eupachyrrhynchus* Heller, 1912. For over a century, this genus remained monotypic, having *E.superbus* Heller, 1912 as the sole representative species. Knowledge about this genus remained stagnant until *Macrocyrtusrukmaneae* Barsevskis, 2016 from Marinduque Island was described and later transferred by Yoshitake and Yap (2017) to the genus *Eupachyrrhynchus*. In the same paper, the authors described another species, *E.badiovittatus* Yoshitake, 2017, from Calayan Island, Cagayan Valley region. Two years later, Rukmane-Bārbale (2019) described four additional species under the genus, namely *E.auromaculatus* Rukmane, 2019 from Cagayan Valley, *E.barbalsi* Rukmane, 2019 from Sierra Madre, *E.barsevskisi* Rukmane, 2019 from Cagayan Valley, and *E.viridimaculatus* Rukmane, 2019 from Isabela Province. To date, the genus *Eupachyrrhynchus* comprises seven species, all within the Greater Luzon Pleistocene Aggregate Island Complex, ranging from Marinduque Island in the south to Calayan Island in the north. In this paper, we describe an additional species of *Eupachyrrhynchus* (the eighth species for the Philippine fauna) and a new species of *Metapocyrtus* Heller, 1912. The paper provides notes on the species’ habitat and mimicry complex, along with high-definition images of their habitus and genitalia.

## ﻿Material and methods

Morphological characters were observed under Leica Luxeo 4D, and Nikon SMZ745T stereomicroscopes. The treatment of the genitals follows Yoshitake (2011). Images of the habitus were taken using a Canon EOS 6D digital camera equipped with a Canon MP-E 65-mm macro lens. Images were stacked and processed using a licensed version of Helicon Focus v. 6.7.0; light and contrast were adjusted in Photoshop CS6 Portable software. Measurements (in mm) and labels mentioned in this paper are abbreviated as follows:

/ different lines;

// different labels;

**LB** body length, from the apical margin of the pronotum to the apex of the elytra;

**LE** elytral length, from the level of the basal margins to the apex of the elytra;

**LP** pronotal length, from the base to apex along the midline;

**LR** length of the rostrum;

**WR** maximum width across the rostrum;

**WE** maximum width across the elytra;

**WP** maximum width across the pronotum.

Comparative materials and specimens used in the study are deposited in the following institutional and private collections:

**CASENT**California Academy of Sciences Entomology Collection, San Francisco, CA, USA;

**CMNC** Canada Museum of Nature, Ottawa, Canada;

**DGC** Daven and Graden Private Collection, Mati, Philippines;

**DUBC** Daugavpils University Beetle Collection, Daugavpils, Latvia;

**MMCP** Milton Medina Collection, Tagum, Philippines;

**PNMNH**National Museum of Natural History Philippine National Museum under Philippine National Museum (**PNM**), Manila, Philippines;

**REQP** Private collection of Randel Estacio, Quezon City University, Quezon City, Philippines;

**SMTD** Senckenberg Natural History Collections, Dresden, Germany;

**TIRL** Terrestrial Invertebrate Research Laboratory, Davao Oriental State University, City of Mati, Philippines

## ﻿Results

### ﻿Taxonomy

#### ﻿ Eupachyrrhynchus joybelmonteae

Taxon classificationAnimaliaColeopteraCurculionidae

Cabras, Estacio, Agbas & Obrial sp. nov.

AE432046-FCC4-50B5-9320-446D6DAB7DF9

https://zoobank.org/11B55DF3-13FC-4B4C-9C41-46B836CE42C8

Figs 1A–D , 3A , 4D

##### Type material.

**Figure 1.**
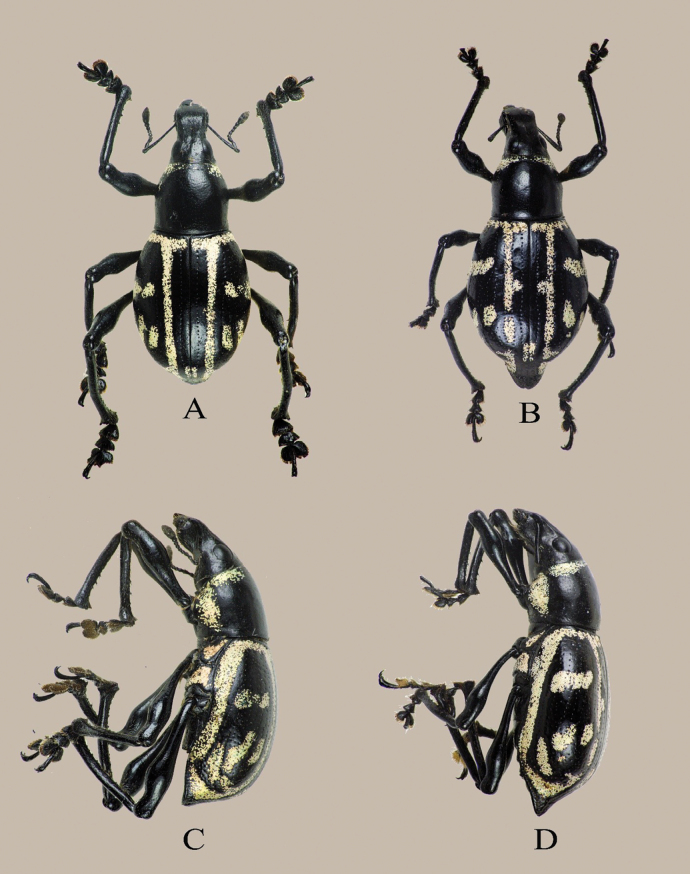
*Eupachyrrhynchusjoybelmonteae* Cabras, Estacio, Agbas & Obrial, sp. nov.: **A.** Male holotype, dorsal habitus; **B.** Female paratype, dorsal habitus; **C.** Male, lateral habitus; **D.** Female, lateral habitus.

***Holotype*** (Fig. 1A, C), • male: “Philippines – Luzon Island, Tanay, Rizal/ 27 iii, 2024 / leg. R. Estacio (typed on white card) // HOLOTYPE male / *Eupachyrrhynchusjoybelmonteae* sp. nov. / CABRAS, ESTACIO, AGBAS & OBRIAL 2025 (typed on red card)” presently in TIRL to be deposited in PNM. ***Paratypes*** • 7 ♂♂, 2 ♀♀ (4♂♂, 1♀ presently at DGC; 3 ♂♂, 1♀ presently at REQP). Same data as holotype.

##### Paratype deposition.

1 ♂ and 1 ♀, DGC; 1 ♂, MMCP; 1 ♂, to be deposited in SNHC; 1 ♂ and 1 ♀, REQP; 1 ♂, currently in REQP, to be deposited in CMN; 1 ♂, currently at REQP, to be deposited in CAS; 1 ♂, currently in DGC, to be deposited in DUBC.

##### Description.

**Male.** Dimensions: LB: 11.5– 2.3 mm (HT: 11.5 mm). LR: 2.1–2.3 mm (HT: 2.1 mm). WR: 1.6–2.0 mm (HT: 1.6 mm). LP: 3.5–3.7 mm (HT: 3.7 mm). WP: 3.3–3.8 mm (HT: 3.8 mm). LE: 8.0–8.5 mm (HT: 8.0 mm). WE: 6.0–6.5 mm (HT: 6.0 mm). *N* = 7.

##### Diagnosis.

**Figure 2.**
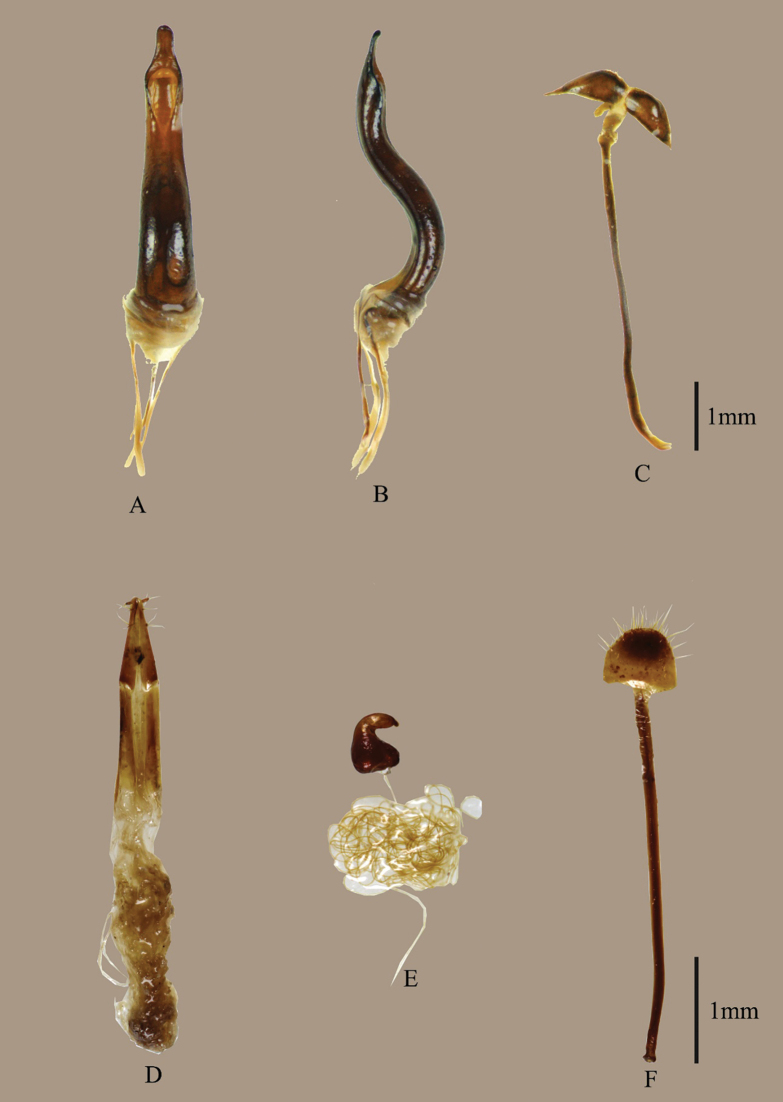
Male genitalia of *Eupachyrrhynchusjoybelmonteae* Cabras, Estacio, Agbas & Obrial, sp. nov. and female terminalia of Metapocyrtus (Metapocyrtus) daraitanensis Cabras, Estacio, Agbas & Obrial, sp. nov.: **A−D.***E.joybelmonteae* sp. nov.: **A.** Aedeagus, dorsal view; **B.** Idem, lateral view; **C.** Sternite IX in dorsal view; **D−F.**Metapocyrtus (Metapocyrtus) daraitanensis Cabras, Estacio, Agbas & Obrial, sp. nov.: **D.** Ovipositor; **E.** Spermatheca; **F.** Sternite VIII.

**Figure 3.**
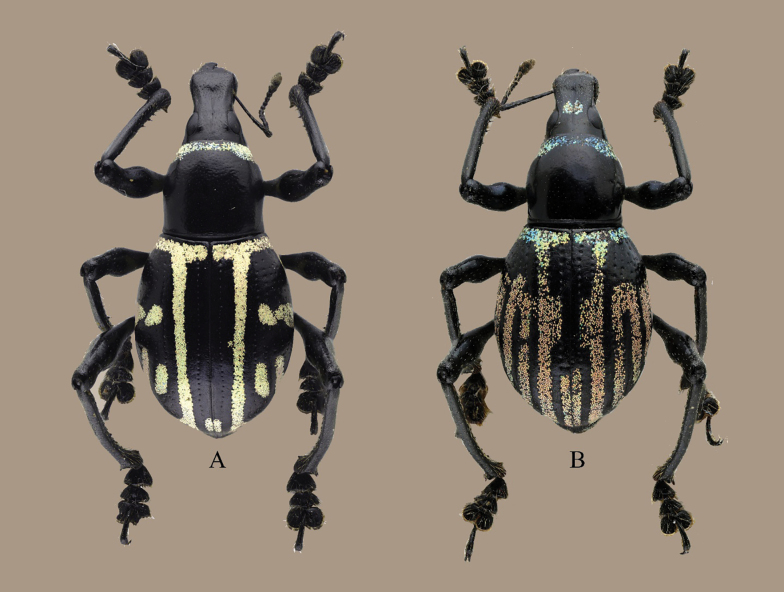
Habitus of *Eupachyrrhynchus* spp. **A.***E.joybelmonteae* Cabras, Estacio, Agbas & Obrial, sp. nov.; **B.***E.rukmaneae*.

*Eupachyrrhynchusjoybelmonteae* sp. nov. closely resembles *E.rukmaneae* Barsevskis, 2016, but can easily be distinguished by its unique elytral markings (Fig. 3) and based on the following characters: rostrum of *E.joybelmonteae* sp. nov. with cordate-shaped depression (as compared to *E.rukmaneae*, with deep, elongated groove-shaped impression); prothorax bell-shaped (as compared to *E.rukmaneae* subspherical); and elytra with fine striae punctures (as compared to *E.rukmaneae*, coarsely striaepunctate). Male genitalia are shown in Fig. 2A−C.

##### Description.

Body medium size (11.5 mm to 12.3 mm). Integuments black. Body surface, rostrum, head, legs, and underside with a weak luster. Head with sparse and minute punctures; forehead between eyes flat; lateroventral side below the eyes with a few sparse appressed piliform and elliptical, peach scales. Eyes weakly convex, almost flattish, not prominent from the outline of head as viewed dorsally. ***Rostrum*** continuous with the head, mostly minutely punctate, some punctures have short setae; almost as long as wide (LR 2.0 mm; WR 1.6 mm); dorsum with a short midline furrow extending until basal half and a shallow cordate-shaped depression; dorsal contour in lateral view almost flat and subapically gradually declined towards apex; lateral contour in dorsal view slightly constricted at base and weakly widened anteriorly, widest at apex; dorsolateral margin oblique, and apex with short yellowish setae. Antennal scape slightly curved, thin at base and slightly widens towards apex with appressed setae. Funicle 7-segmented subequal to scape, covered with suberect setae. Antennal segment I slightly longer than II, three times longer than wide; segment III slightly longer than IV-VI, slightly longer than wide; segments 4–6 nearly as long as wide, and segment 7 slightly wider than long, wider than segments III-VI; club sub-ellipsoidal, nearly three times longer than wide. ***Prothorax*** sub-cylindrical as viewed dorsally, basal margin wider than apical margin, as long as wide (LP 3.7 mm; WP 3.8 mm), mostly smooth with very minute punctures and scaly marks on the apical and lateral margin. Pronotum with the following scaly marking of round, appressed, and contiguous pale-yellow ochre scales: a) thin stripes along apical margin, and b) thin stripe along lateral margin before coxae. ***Elytra*** ovate, longer than wide (LE 8.0 mm / WE 6.0 mm), wider and twice longer than prothorax (WE 6.0 mm; WP 3.8 mm; LE 8.0 mm; LP 3.7 mm), body surface striate punctate with punctures distinct and evenly spaced from each other. Elytra with the following scaly markings of round, appressed, and contiguous pale yellow ochre scales: a) thin stripe at basal margin, b) longitudinal stripe on interval 3 extended from base to apex, c) thin stripe along lateral margin, confluent with the basal stripe and on interval 3 along middle, d) two small premedial patches on dorsolateral surface, e) three short stripes at the apicad on dorsolateral surface, one short subdorsal, one medium size on dorsolateral surface, and one just above the lateral margin, and f) small dots along suture at the base of apical declivity. Dorsal contour in lateral view flattish with abrupt apical declivity, lateral contour widened as viewed dorsally, uniformly arcuate on sides, widest at middle.

***Legs*** with moderately clavate femora. Femora black, sparsely covered with minute setae. Tibiae covered with appressed, minute setae on external surface; internal margin with brown suberect setae. Tibiae denticulated and mucronate at apex. Tarsi with moderately long suberect and sparse setae; tarsomere 1 subtriangular, wider than long, nearly as long as tarsomere 2; tarsomere 2 short, narrowly subtriangular, almost 2.5× wider than long; tarsomere 3 bilobed, subequal in length with tarsomere 1, longer than tarsomere 2; tarsomere 5 longer than tarsomere 1; tarsal claws free.

***Coxae*** with sparse, very minute, appressed setae. Mesoventrite with sparse, very minute, appressed setae; mesepesternite with a patch of appressed, imbricate, and peach scales; metaventrite with sparse and minute setae and a patch of appressed, imbricate, pale-yellow ochre scales on distal ends. Ventrite I and II connate at middle; Ventrite II weakly convex, III-V well separated; Ventrite I-V with minute pubescence; and Ventrite V flat and with minute punctures and short pubescence posteriorly. Male genetalia and sterninte IX (Fig. 2A−C).

**Female.**LB: 11.1 mm – 13.5 mm. LR: 1.7 mm – 2.2 mm. WR: 1.5 mm. LP: 3.8 mm – 4.0 mm. WP: 3.5–4.0 mm. LE: 8.0 mm – 9.5 mm. WE: 5.7 mm – 6.5 mm. *N* = 2. Habitus as shown in Fig. 1B, D. Female is very similar to the male but differs in the following characteristics: a) Ventrite 1 and 2 connate throughout, lustrous, and convex, b) Ventrite V convex at middle with depression on sides, c) apex of Ventrite 5 sub-hemispherical, and d) apex of elytra with projection, tarsi different as per genus character.

##### Etymology.

*Eupachyrrhynchus joybelmonteae* Cabras, Estacio, Agbas & Obrial, sp. nov. is named after the 11^th^ mayor of Quezon City, Hon. Mayor Maria Josefina Tanya “Joy” Go Belmonte. She has been named one of the 2023 Champions of the Earth by the United Nations Environment Programme (UNEP) for her leadership and policy achievements in curbing plastic pollution and greening Quezon City.

##### Distribution.

*Eupachyrrhynchusjoybelmonteae* Cabras, Estacio, Agbas & Obrial, sp. nov. is known so far from its type locality in Tanay, Rizal.

#### ﻿ Metapocyrtus (Metapocyrtus) daraitanensis

Taxon classificationAnimaliaColeopteraCurculionidae

Cabras, Estacio, Agbas & Obrial sp. nov.

7CDFCA90-CE67-5B5F-9866-BF326B1B91E2

https://zoobank.org/489AC77C-212C-4ED6-8300-3D09B93A39C7

Figs 3A, B , 4A

##### Type material.

***Holotype*** (Fig. 1A, B), • female: “Philippines – Luzon Island, Tanay, Rizal, Brgy. Daraitan/ 27 iii, 2024 / leg. R. Estacio (typed on white card) // HOLOTYPE female / Metapocyrtus (Metapocyrtus) daraitanensis sp. nov. / CABRAS, ESTACIO, AGBAS & OBRIAL 2025 (typed on red card),” presently in TIRL to be deposited in PNM.

##### Description.

**Female.** Dimensions: LB: 9.5 mm. LR: 1.8 mm. WR: 1.4 mm. LP: 2.7 mm. WP: 3.5 mm. LE: 6.3 mm. WE: 5.3 mm. *N* = 1.

##### Diagnosis.

Metapocyrtus (Metapocyrtus) daraitanensis sp. nov. superficially resembles M. (M.) mandarinus Heller, 1916, and M. (M.) pseudomandarinus Heller, 1921, but differs in the following characteristics: eyes closer together, not prominent on outline of head (as compared to M. (M.) mandarinus further apart, situated at sides; M. (M.) pseudomandarinus convex, weakly prominent on outline of head); prothorax weakly arched dorsal contour, with two slightly angled scaly markings on each side of disc (as compared to M. (M.) mandarinus, prominently arched dorsal contour, with two sub-falcate scaly markings; M. (M.) pseudomandarinus, weakly broadly arcuate dorsal contour, with two slanted longitudinal scaly markings); further, as compared to M. (M.) pseudomandarinus with a weakly granulate projection of surface of prothorax, M. (M.) daraitanensis has a smoother projection; elytra with four closely arranged horizontally spots at apical declivity (absent from both M. (M.) mandarinus and M. (M.) pseudomandarinus); and surface between striael punctures even (as compared to M. (M.) pseudomandarinus with an uneven projection). Female terminalia shown in Fig. 2D−F.

##### Description.

***Coloration***: Integuments, black except antennae reddish brown; Body surface, rostrum, head, legs, and underside with a weak luster. Head with sparse and minute punctures; forehead covered with sparse, shallow punctures, flat, weakly convex anteriorly; lateral surface below eye with imbricate, appressed, elliptical, peach scales interspersed with sparse piliform scales same coloration. Eyes medium-sized, matte, feebly convex, and moderately prominent from outline of head.

***Rostrum*** separated from the head by a distinct transverse groove, sparsely covered with fine punctures longer than wide (LR 1.8 mm; WR 1.3 mm); dorsal surface with a scaly patch on basal half with appressed, ovate, and peach scales; lateral sides with sparse and suberect setae near apex; dorsum with a weakly depressed midline furrow extending until basal two-thirds; dorsal contour in lateral view almost flat and subapically gradually declined towards apex; lateral contour in dorsal view slightly constricted at base and weakly widened anteriorly, widest at apex; dorsolateral margin rounded, and apex with yellowish setae. Scape and funicle setose subequal in length; scape covered with appressed setae, funicle covered with suberect setae. Antennae with segments I and II subequal in length, three times longer than wide; segments III to IV slightly longer than wide, longer than segments V to VII; segments V-VI subequal, and segment VII slightly wider than long; club sub-ellipsoidal, nearly three times longer than wide. ***Prothorax*** sub-globular, basal margin wider than apical margin, wider than long (LP 2.7 mm; WP 3.5 mm), mostly smooth with sparse and minute punctures, each puncture with very minute setae, and scaly marks. Pronotum with the following scaly marking of round, appressed, and imbricate peach scales: thin stripes along apical and lateral margins, and two longitudinal recurved stripes on each side of pronotum; dorsal contour in lateral moderately convex, lateral contour in dorsal view from base to apex uniformly arcuate, widest on apical third. ***Elytra*** ovate, longer than wide (LE 6.3 mm / WE 5.3 mm), wider and more than twice longer than prothorax (WE 5.3 mm; WP 3.5 mm; LE 6.3 mm; LP 2.7 mm). Each elytron striate punctate with the following scaly markings of round, appressed and imbricate peach scales: a) thin stripe near base, b) one longitudinal continuous thin stripe from base to apex, subparallel to each other dorsally, c) thin transverse stripe along middle, thin stripe along lateral margin contiguous with basal and dorsal stripe forming a triangular scaly mark, d) two thin longitudinal stripes on lateral sides interrupted on apicad, and e) small spots near suture subapically almost forming a thin stripe. Dorsal contour in lateral view strongly convex with gradual apical declivity, lateral contour in dorsal view uniformly convex, gradually widened from a narrow base towards middle, then gradually narrows towards a weakly acute apex, widest at middle.

**Figure 4.**
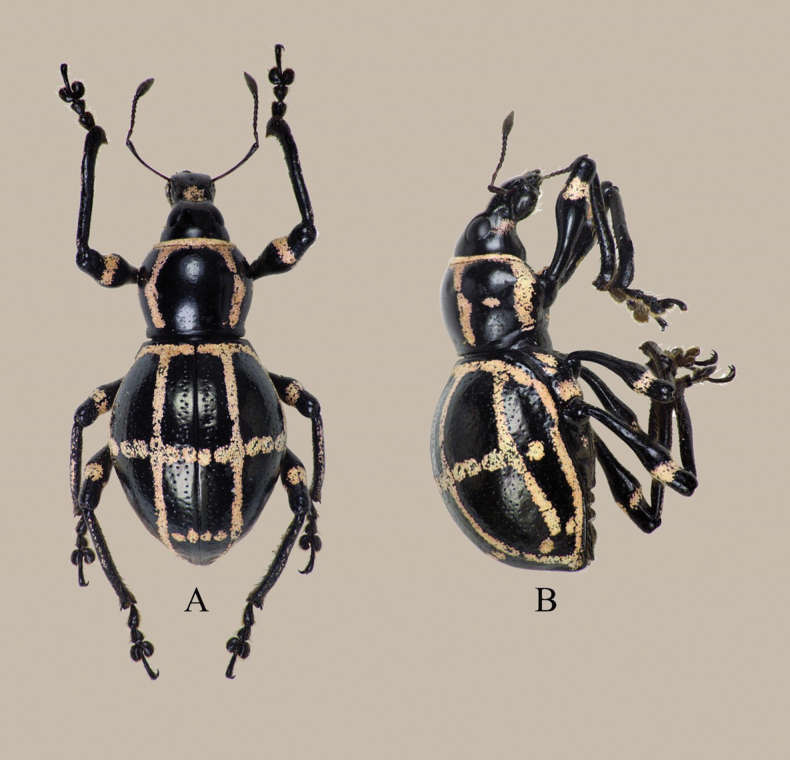
Habitus of Metapocyrtus (Metapocyrtus) daraitanensis Cabras, Estacio, Agbas & Obrial, sp. nov. **A.** Dorsal; **B.** Ditto, lateral.

***Legs*** with moderately clavate femora. Femora black, sparsely covered with minute setae and with a band of appressed, round and contiguous peach scales on apical third. Tibiae covered with subappressed, brown setae, and with appressed, round to elliptical, contiguous peach scales on external margin. Protibiae with denticles; pro and mesotibiae mucronate at apex. Tarsi is moderately long with long suberect setae, mesotarsomere 1, and metatarsomere 1 subequal, slightly longer than protarsomere 1. Tarsomere 1 elongate, and narrowly subtriangular, longer than tarsomere 2; tarsomere 2 short, triangular, and simple without sharp projections at apical corners; tarsomere 3 bilobed, subequal in length with tarsomere 2; tarsomere 5 longer than tarsomere 1; tarsal claws free. Coxae covered with sparse, suberect setae. Mesoventrite covered with short pubescence; mesepesternite with a patch of appressed, imbricate and peach scales; metaventrite with short pubescence and a patch of appressed, imbricate, pale yellow and peach scales on distal ends; Ventrite I and II connate and weakly convex, III-V well separated. Ventrite I-V with minute pubescence. Ventrite V weakly convex anteriorly and with small punctures posteriorly.

**Male.** Unknown.

##### Etymology.

*Metapocyrtus* (*Metapocyrtus*) *daraitanensis* Cabras, Estacio, Agbas & Obrial, sp. nov. is named after its type locality, Barangay Daraitan in Tanay, Rizal, Philippines, where the species was discovered.

##### Distribution.

Metapocyrtus (Metapocyrtus) daraitanensis Cabras, Estacio, Agbas & Obrial, sp. nov. is known so far from its type locality in Tanay, Rizal.

### ﻿Notes on mimicry

**Figure 5.**
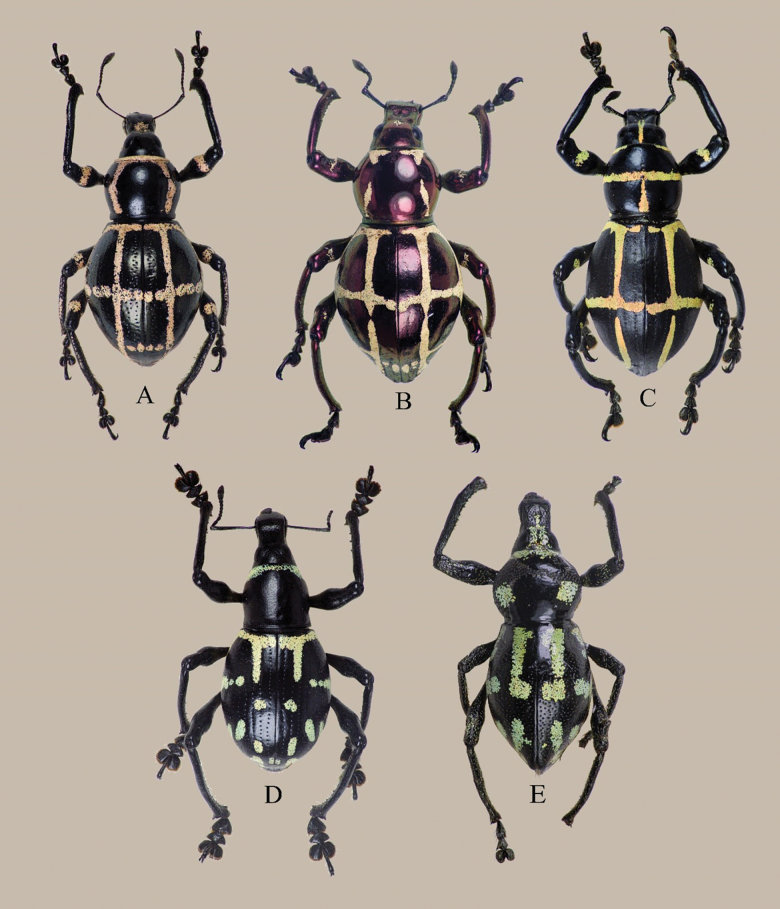
Mimicry complex: **A.**Metapocyrtus (Metapocyrtus) daraitanensis Cabras, Estacio, Agbas & Obrial, sp. nov.; **B.***Pachyrhynchusrochaorum* Yoshitake, 2020; **C.***P.moniliferus* Germar, 1824; **D.***E.joybelmonteae* Cabras, Estacio, Agbas & Obrial, sp. nov.; **E.***Metapocyrtus* sp.

The species described in this paper exhibits Mullerian mimicry with other species under different genera from the tribe Pachyrhynchini. Mullerian mimicry is characterized by different species having similar appearances, a tactic that reduces predation by training predators to avoid the patterns/colorations they display (Sherratt 2008; Agbas et al. 2024; Obrial et al. 2024). Mimicry among the tribe Pachyrhynchini has been well-documented since Schultze’s (1923, 1925) time, where species found within the same locality exhibit remarkable similarities in coloration and markings. More often, these mimicking species belong to different genera or families within Coleoptera, such as the well-known longhorn beetle in the genus *Doliops*, or even entirely different insect orders. In addition to their hardened bodies, which protect against accidents caused by unpredictable weather conditions, these weevils’ coloration is not merely decorative but plays a crucial role in their survival (Schultze 1923). The conspicuous coloration also serves as an aposematic signal, warning predators of their unpalatability (Tseng et al. 2014; Van Dam et al. 2024). One of the earliest studies on mimicry within the tribe was made by Van Dam et al. (2023), who proposed that species in the genus *Pachyrhynchus* exhibit frequency-dependent mimicry, suggesting that when a particular color pattern or phenotype is numerically dominant within a population, it is favored by natural selection because it enhances predator learning and avoidance. For instance, a similar mimicry complex has been observed in *Metapocyrtusinangsabong* Cabras, Obrial & Agbas, 2024, which shares mimetic colorations and patterns with other species in the genus and with other beetle species found in the same locality (Medina et al. 2024; Obrial et al. 2024). The two new species described here—*Eupachyrrhynchusjoybelmonteae* sp. nov. and Metapocyrtus (Metapocyrtus) daraitanensis sp. nov.—appear to be part of a Mullerian mimetic ring in the area. This ring includes *Pachyrhynchusrochaorum*, *Pachyrhynchusmoniliferus*, and *Metapocyrtus* sp., all of which exhibit similar elytral markings characterized by transverse and longitudinal stripes as shown in Fig. 5.

### ﻿Ecologic notes

**Figure 6.**
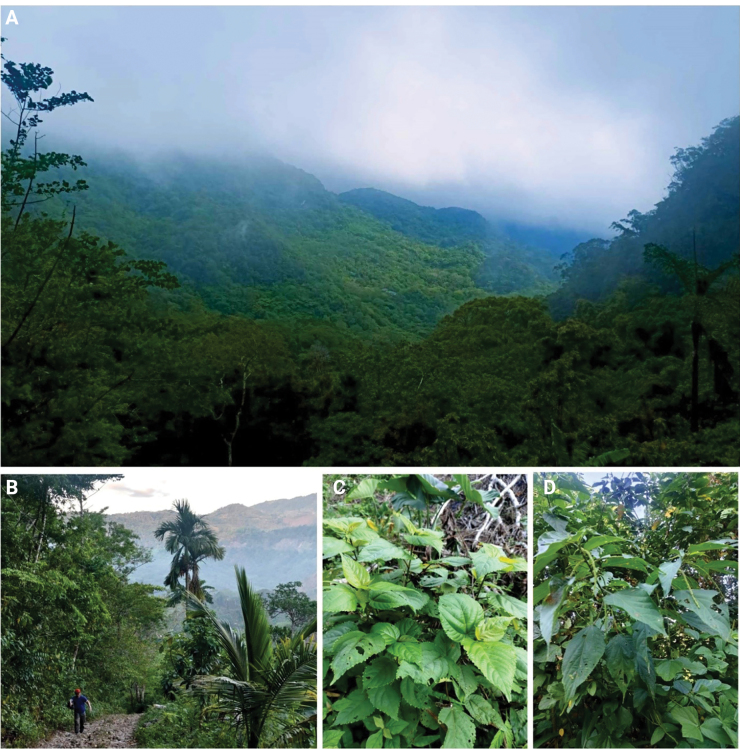
**A.** pristine forest in Barangay Daraitan, Tanay Rizal, going to Genral Nakar, Quezon, Philippines; **B.** trail from where the specimens were collected; **C.**Acalyphacf.cardiophylla (Euphorbiaceae); **D.***Acalyphaamentacea*.

*Eupachyrrhynchusjoybelmonteae* sp. nov. and M. (M.) daraitanensis sp. nov., were found along a forested trail within a mountainous area in Barangay Daraitan, Tanay Rizal, going to General Nakar, Quezon, Philippines, at an elevation of approximately 450 to 500 m above sea level. The locality is characterized by dense vegetation and a well-structured forest canopy with an understory composed of various ferns, shrubs, and native flowering plants. The specimens were observed on specific host plants, with *E.joybelmonteae* collected from the leaves of Acalyphacf.cardiophylla (Fig. 6C) and M. (M.) daraitanensis on *Acalyphaamentacea* (Fig. 6D).

The habitat of the species faces increasing environmental pressures. The expansion of human activities such as agricultural land conversion and small-scale logging poses a direct threat to the integrity of the forest. Fragmented clearing has led to the introduction of invasive plant species and increased soil erosion, which further destabilizes the ecosystem. Additionally, infrastructure development in nearby areas facilitates easier access to the forest, raising concerns about potential disturbances, including poaching and resource exploitation.

To ensure protection of these species and their habitat, conservation efforts must prioritize habitat preservation, sustainable land management, and community engagement. Scientific research continues to provide critical insights into the biodiversity of the region, emphasizing the need for long-term monitoring and conservation initiatives. Protecting these forested areas is vital to maintaining ecological balance and safeguarding species that are yet to be fully documented.

## Supplementary Material

XML Treatment for Eupachyrrhynchus joybelmonteae

XML Treatment for Metapocyrtus (Metapocyrtus) daraitanensis

## ﻿Additional information

### Use of AI

No use of AI was reported.

### Author ORCIDs

Analyn A. Cabras https://orcid.org/0000-0002-0980-1651

Randel D. Estacio https://orcid.org/0000-0002-6943-0493

Daven Jayson D. Agbas https://orcid.org/0009-0005-0862-5181

Graden G. Obrial https://orcid.org/0009-0004-4163-6942

### Data availability

All of the data that support the findings of this study are available in the main text.
